# Effect of Digital Early Warning Scores on Hospital Vital Sign Observation Protocol Adherence: Stepped-Wedge Evaluation

**DOI:** 10.2196/46691

**Published:** 2024-06-20

**Authors:** David Chi-Wai Wong, Timothy Bonnici, Stephen Gerry, Jacqueline Birks, Peter J Watkinson

**Affiliations:** 1 Leeds Institute of Health Sciences School of Medicine University of Leeds Leeds United Kingdom; 2 Critical Care Division University College Hospital London NHS Foundation Trust London United Kingdom; 3 Centre for Statistics in Medicine University of Oxford Oxford United Kingdom; 4 Oxford University Hospitals NHS Trust Oxford United Kingdom; 5 NIHR Biomedical Research Centre Oxford University Hospitals NHS Foundation Trust Oxford United Kingdom; 6 Nuffield Department of Clinical Neurosciences Kadoorie Centre for Critical Care Research and Education University of Oxford Oxford United Kingdom

**Keywords:** vital signs, early warning score, track and trigger, electronic charting, stepped-wedge, vital, charting, documentation, deterioration, hospital management, clinical intervention, decision-making, patient risk, hospital, ICU, intensive care unit, UK, United Kingdom, intervention

## Abstract

**Background:**

Early warning scores (EWS) are routinely used in hospitals to assess a patient’s risk of deterioration. EWS are traditionally recorded on paper observation charts but are increasingly recorded digitally. In either case, evidence for the clinical effectiveness of such scores is mixed, and previous studies have not considered whether EWS leads to changes in how deteriorating patients are managed.

**Objective:**

This study aims to examine whether the introduction of a digital EWS system was associated with more frequent observation of patients with abnormal vital signs, a precursor to earlier clinical intervention.

**Methods:**

We conducted a 2-armed stepped-wedge study from February 2015 to December 2016, over 4 hospitals in 1 UK hospital trust. In the control arm, vital signs were recorded using paper observation charts. In the intervention arm, a digital EWS system was used. The primary outcome measure was time to next observation (TTNO), defined as the time between a patient’s first elevated EWS (EWS ≥3) and subsequent observations set. Secondary outcomes were time to death in the hospital, length of stay, and time to unplanned intensive care unit admission. Differences between the 2 arms were analyzed using a mixed-effects Cox model. The usability of the system was assessed using the system usability score survey.

**Results:**

We included 12,802 admissions, 1084 in the paper (control) arm and 11,718 in the digital EWS (intervention) arm. The system usability score was 77.6, indicating good usability. The median TTNO in the control and intervention arms were 128 (IQR 73-218) minutes and 131 (IQR 73-223) minutes, respectively. The corresponding hazard ratio for TTNO was 0.99 (95% CI 0.91-1.07; *P*=.73).

**Conclusions:**

We demonstrated strong clinical engagement with the system. We found no difference in any of the predefined patient outcomes, suggesting that the introduction of a highly usable electronic system can be achieved without impacting clinical care. Our findings contrast with previous claims that digital EWS systems are associated with improvement in clinical outcomes. Future research should investigate how digital EWS systems can be integrated with new clinical pathways adjusting staff behaviors to improve patient outcomes.

## Introduction

Avoidable mortality from unrecognized clinical deterioration is an internationally recognized problem [1]. Such deterioration often corresponds with deviations in patient vital signs early warning score (EWS) algorithms have been introduced to improve the recognition of abnormal vital signs [2]. They assign a score to each vital sign value according to the degree of abnormality. The total score is a measure of patient risk. Many EWS algorithms have been published and their use is mandated by the National Institute of Health and Care Excellence in the United Kingdom [3,4]. Since 2018, 1 standard EWS, the National Early Warning Score 2, has been mandated in acute hospital trusts [5].

EWS algorithms are accompanied by an escalation protocol, which dictates how frequently the patient should be monitored and what other actions staff should take for each value of the total score. If the EWS score exceeds the “trigger threshold” defined in the escalation protocol, the nursing staff must call a doctor to review the patient.

Despite the widespread adoption of EWS algorithms and associated escalation protocols, patient outcomes have not improved significantly [6,7]. It is possible that errors in the calculation of the EWS are partially to blame. Studies have shown that errors in the calculation of EWS are common and failure to calculate the correct EWS may result in failure to take the correct action [8,9]. Other barriers to escalation include delays in documentation, lack of familiarity with the escalation protocol, failure to follow the protocol, and poor communication [10,11].

Digital EWS systems have been proposed as a solution. These systems automatically calculate the EWS based on data input by staff and display relevant information from the escalation protocol. These data may be displayed to the staff at the bedside, on mobile devices, or at nursing station dashboards, enabling senior clinicians to rapidly survey patient acuity across an area.

At present there is no robust evidence of changes in clinical outcomes to support or refute the case for the introduction of electronic EWS systems. Most recent studies focus on improving the predictive ability of the scoring system itself [12,13], ignoring the complex interaction with health care staff and infrastructure required to affect clinical decision-making. The limited number of studies of digital EWS systems in clinical practice have shown inconsistent results [14-16]. Some have used uncontrolled “before and after” design methodologies, comparing data from periods several years apart, which are limited by their inability to control for temporal confounding such as changes in case mix [17]. Furthermore, very few existing studies have not provided insight into the mechanisms by which any reported improvements were achieved [18].

This study aimed to examine whether the introduction of a digital EWS charting system leads to improvements in patient care. Our causal hypothesis is that, compared with paper charting, the use of a digital EWS system leads to better recognition of patient deterioration and closer adherence to the hospital escalation protocol. These behavior changes would lead to the more frequent observation of patients with abnormal vital signs and therefore earlier escalation. Earlier escalation would lead to improvements in both process metrics and patient outcomes.

## Methods

The staged replacement of paper EWS charting with a digital EWS charting system at the Oxford University Hospitals Foundation NHS Trust (OUHFT) provided us the opportunity to conduct a natural experiment using a nonrandomized stepped wedge trial design.

### Ethical Considerations

The study protocol was reviewed by the OUHFT’s Research and Development department, and based upon Health Care Quality Improvement Partnership guidelines and was deemed to be a service evaluation (ID: 3196), not requiring review by the National Research Ethics Service. All methods were carried out in accordance with the Declaration of Helsinki. As patient data were collected without their consent, permission for informed consent waiver was obtained from the Trust’s Caldicott Guardian and Medical Director in accordance with the Health Research Authority Confidentiality Advisory Group guidelines. All study data were deidentified and patients were not compensated. The full study protocol has previously been published and is summarized below [19].

### Study Setting

The OUHFT is comprised of 4 hospitals: 1 large teaching hospital, a small district general hospital, and 2 specialist hospitals that do not have emergency departments. Two of the hospitals have intensive care units (ICU) that also act as high-dependency units, and 2 have high-dependency units only.

The digital EWS implemented at OUHFT was the system for electronic notification and documentation (SEND) system [20], a system in which clinical users manually enter vital sign observation data onto a tablet PC. The system then automatically calculates an EWS and displays relevant advice from hospital escalation protocols.

**Figure 1:**
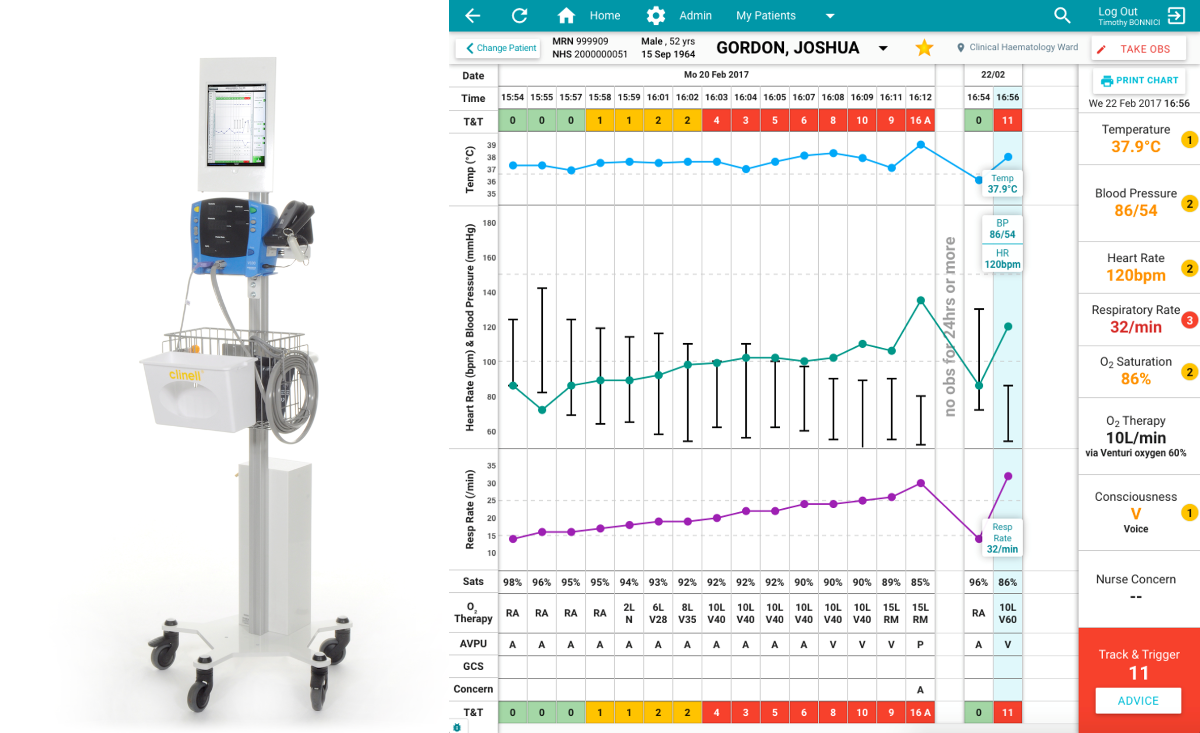
Overview of the system for electronic notification and documentation (SEND) digital early warning score (EWS) system. The left image shows how SEND is run on a tablet PC, housed alongside the equipment required to take a set of vital sign observations and a barcode scanner to positively identify the patient and clinician. The right image shows a screenshot from SEND, once a patient has been positively identified. Historical vital signs are presented in a familiar format, and an option to document a new set of observations is available via the top-right “Take Obs” button. Further details of this system are provided by Wong et al [20].

The tablet is physically mounted to a roll-stand with a blood pressure monitor, as shown in Figure 1. The system displays historical vital sign observations of a patient (Figure 1), and ward-level and hospital-level overviews are available via desktop computers.

The EWS used at OUHFT was the centile early warning score (CEWS) [21]. CEWS uses 6 vital signs as input parameters, which are each scored from 0 to 3 (Table 1). The trigger threshold is set at 3. For any CEWS greater than or equal to the trigger threshold, the escalation protocol mandates hourly observations and review by a senior doctor. CEWS also allows a nurse to indicate clinical concern. When a nurse is concerned, hourly observations and escalation to a doctor are mandated, irrespective of the CEWS score. A copy of the paper EWS chart and a full description of the escalation protocol are provided in Multimedia Appendix 1.

**Table 1:** The centile early warning score (CEWS) algorithm used by clinicians at the Oxford University Hospitals Trust to identify patients at risk of deterioration. The subscores for each vital sign are tallied to generate the total EWS. Hospital protocol dictates that a score of 3 or greater warrants senior clinical review, and a new set of observations within 1 hour.

Vital signs	Subscores						
	3	2	1	0	1	2	3
Temperature (°C)	≤35.4	—^a^	35.5-35.9	36.0-37.3	37.4-38.3	—	≥38.4
Heart rate (/min)	≤42	43-49	50-53	54-104	105-112	113-127	≥128
Systolic blood pressure (mm Hg)	≤85	86-96	97-101	102-154	155-164	165-184	≥185
Respiratory rate (/min)	≤7	8-10	11-13	14-19	20-21	22-24	≥25
SpO_2_^b^ (%)	≤84	85-90	91-93	≥94	—	—	—
Level of consciousness (AVPU^c^ or GCS^d^)	P and U GCS ≤13	—	V GCS:14	A GCS: 15	—	—	—

^a^Not available.

^b^SpO_2_: peripheral arterial oxygen saturation.

^c^AVPU: Alert, Voice, Pain, Unresponsive.

^d^GCS: Glasgow coma scale.

### Trial Design

The stepped-wedge study comprised 2 arms, a control arm in which vital signs were recorded using paper observation charts and an intervention arm where the digital EWS system, SEND, was used. The EWS and escalation protocol were identical in both arms.

The study consisted of 20 clusters (and 21 steps). We defined a cluster as a group of between 1 and 5 eligible wards that implemented SEND simultaneously. All wards that were due to switch to the SEND system were eligible for inclusion in a cluster; we defined these as “study wards.” Study wards included all adult wards across the Trust, except for the obstetric wards, emergency departments, day units, high dependency units, ICUs, and investigation suites, which were excluded as they did not use standard hospital observation recording and escalation policies. We also excluded the 3 wards where the SEND system was initially developed and piloted, as the control condition, paper charting, was no longer used at the commencement of the study.

Clusters of wards were determined by pragmatic considerations related to the safe conduct of the rollout. For example, each cluster only contained wards from an individual hospital. The sequence of study clusters was predetermined by the system rollout strategy and was therefore not randomized.

**Figure 2:**
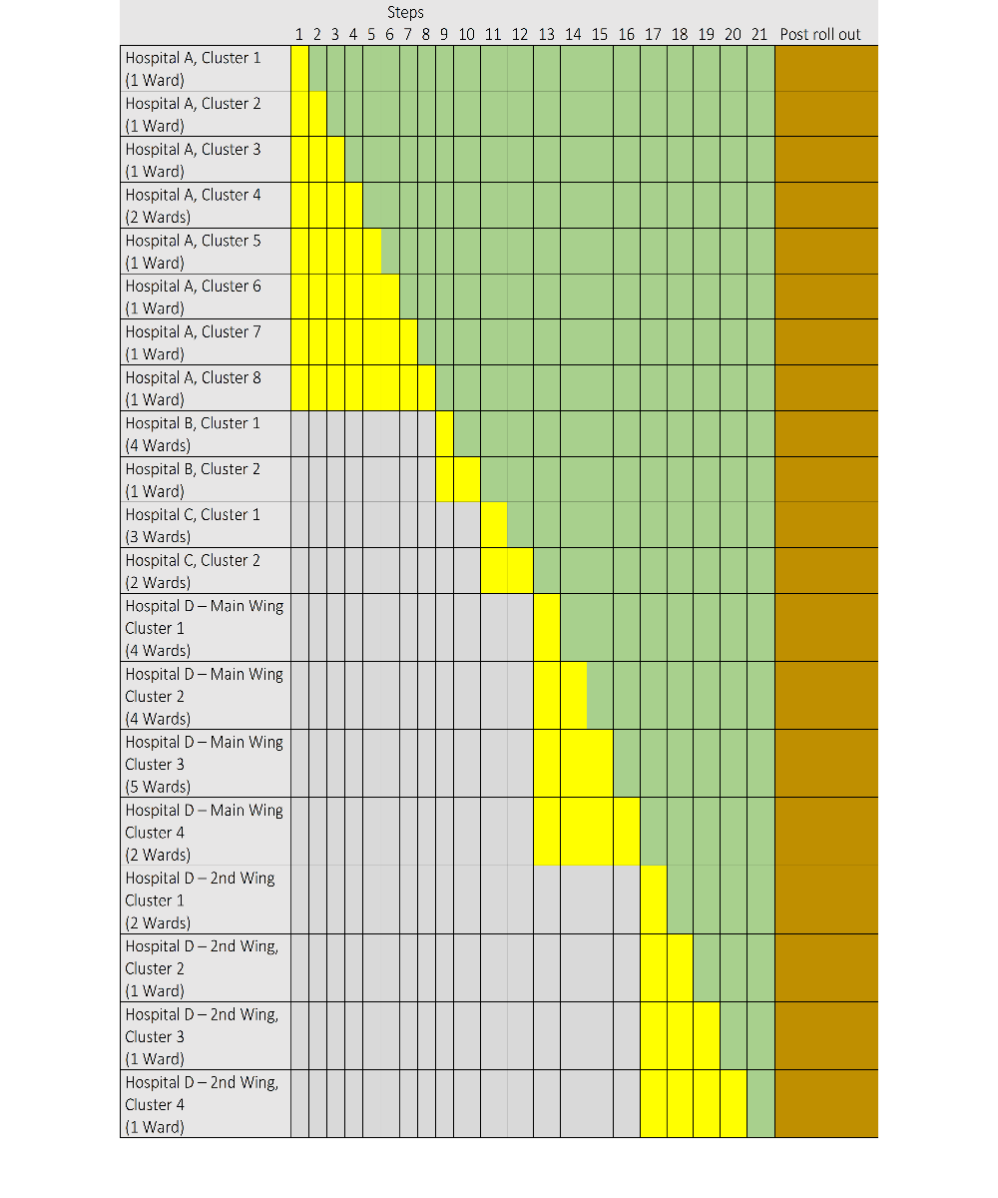
Stepped-wedge data collection with respect to the roll-out schedule. Gray represents periods where no study data were collected, yellow represents periods where data were collected for the control arm, green represents periods where study data were collected for the intervention arm, and brown represents the final 3-month post–roll-out during which study data were collected for all clusters and wards. Ward denotes that the system for electronic notification and documentation (SEND) was deployed to an individual ward. Cluster denotes that SEND was deployed on multiple wards simultaneously.

The rollout schedule is depicted in Figure 2. The time period between the start of each step was typically 2 weeks. The period was occasionally lengthened to account for project management issues such as reduced staffing over the Christmas holidays (exact dates are provided in Multimedia Appendix 2). The final period, which occurred after SEND was fully deployed to all wards, lasted 3 months. The extended period was designed to capture any delayed effects caused by wards adapting to the new system.

Each study ward admitted multiple patients during each step. Data for this study was obtained at an individual patient level. A patient’s data belonged to only 1 step, that is, each cluster and period contained data pertaining to different people. We included all patient admissions to the study wards during the study period rather than censoring data from repeated admissions. Therefore, some patients could potentially contribute data to multiple steps on different admissions. We treated multiple episodes within the same patient as independent, reasoning that the primary outcome was unlikely to be causally related to patient characteristics. We excluded data from admissions where patients crossed study arms (ie, the ward moved from paper to digital EWS) during their admission.

### Data Collection

Data from the control arm were collected by 7 research assistants transcribing data from paper charts located on each study ward into a bespoke electronic form. This was a resource-intensive process, making it unfeasible to collect data from all clusters simultaneously for the duration of the study. Therefore, we commenced data for the control arm at the start of the roll-out to each hospital site and limited it to the site where SEND was actively rolled out (illustrated in Figure 2). To make this tractable, we further split the largest hospital (Hospital D), into 2 sites (Main Wing, second Wing). Data from the intervention arm was continued even once the roll-out of the intervention at a given hospital was complete such that patients from the hospital contributed more data to the intervention arm than data in subsequent hospitals. In summary, data collection may be considered as separate stepped wedges associated with each of the 5 sites, with varying lengths of data from after the intervention.

For each patient admission within each study cluster, we collected patient characteristics (age, gender, Charlson score, admission type, and admitting specialty), the date and times of admission to the ward; first observation with CEWS ≥3 and the immediate subsequent observation; hospital discharge; hospital mortality; transfer to ICU; cardiac arrest call; and theatre admission.

### Outcome Measures

The primary outcome measure was the time to next observation (TTNO), defined as the time between a patient’s first triggering observations set (CEWS score ≥3) and the subsequent observations set. To address potential confounding by length of ward stay, analysis of the primary outcome measure was restricted to triggering observation sets that occurred within 48 hours of transfer to the first study ward of an admission.

Secondary outcome measures were time to death in the hospital, time to unplanned ICU admission, time to cardiac arrest call, and hospital length of stay (LOS). In each case, the start time was the time of the initial triggering set of observations.

We reported these outcomes for the subgroup included in the analysis of the primary outcome measure (ie, those patients who had a CEWS score ≥3), in line with our causal hypothesis. We also reported the secondary outcomes for all eligible admissions. In these analyses, we used the time of admission to the study ward as the start time.

Finally, we reported system usability to provide further context. System usability was measured using the system usability scale, a validated 10-item questionnaire that is used to generate a score between 0 and 100 [22]. We delivered the questionnaire electronically to all users of the digital system. The questionnaire is included in Multimedia Appendix 3.

### Sample Size

The upper bound on the number of patient admissions included in the study was determined by the pragmatic roll-out schedule of the intervention. To determine whether this would be sufficient, we initially undertook a power calculation for steps 1-8, using unpublished pilot data from the Computer Alerting Monitoring System 2 study [23]. We assumed that the proportion of patients who have a further observation within 3 hours of recording an EWS ≥3 would be 0.5 in the paper arm and 0.6 in the electronic arm, that there would be an average of 11 patients with an initial CEWS ≥3 per cluster, and conservatively that the intracluster correlation will be 0.15. The power was then estimated to be 79.3% for a 5% α level. While the calculation depended on statistics estimated from limited pilot data, it indicated that the inclusion of all steps would be sufficiently powerful to detect a difference of 10% in the primary outcome between groups. Full details of this calculation are provided in Multimedia Appendix 4.

### Statistical Methods

The primary outcome, the difference in TTNO between arms, was analyzed using a mixed-effects Cox model with a random intercept for cluster and a fixed effect for time as described by Hussey and Hughes [24]. The model included in-hospital death, ICU admission, theatre admission, and cardiac arrest calls as competing events.

We conducted a sensitivity analysis using 5 variants of the basic Hussey and Hughes model, as originally proposed by Hemming et al [25]. The five variants were: (1) time by strata interaction (fixed effects), (2) time by cluster interaction (random effects), (3) treatment by strata interaction (fixed effects), (4) treatment by cluster interaction (random effects), and (5) treatment by time interaction (fixed effects). Secondary outcomes were analyzed using the same method.

To aid interpretation, we calculated the average TTNO in each arm as the mean of the median (IQR) TTNO within each unit of the stepped wedge cluster.

We reported baseline descriptive statistics on patient characteristics, including age and sex, by study arm. We also reported these data for each time period to help understand whether trends in baseline characteristics differed between the control and intervention arms.

## Results

### Overview

**Figure 3:**
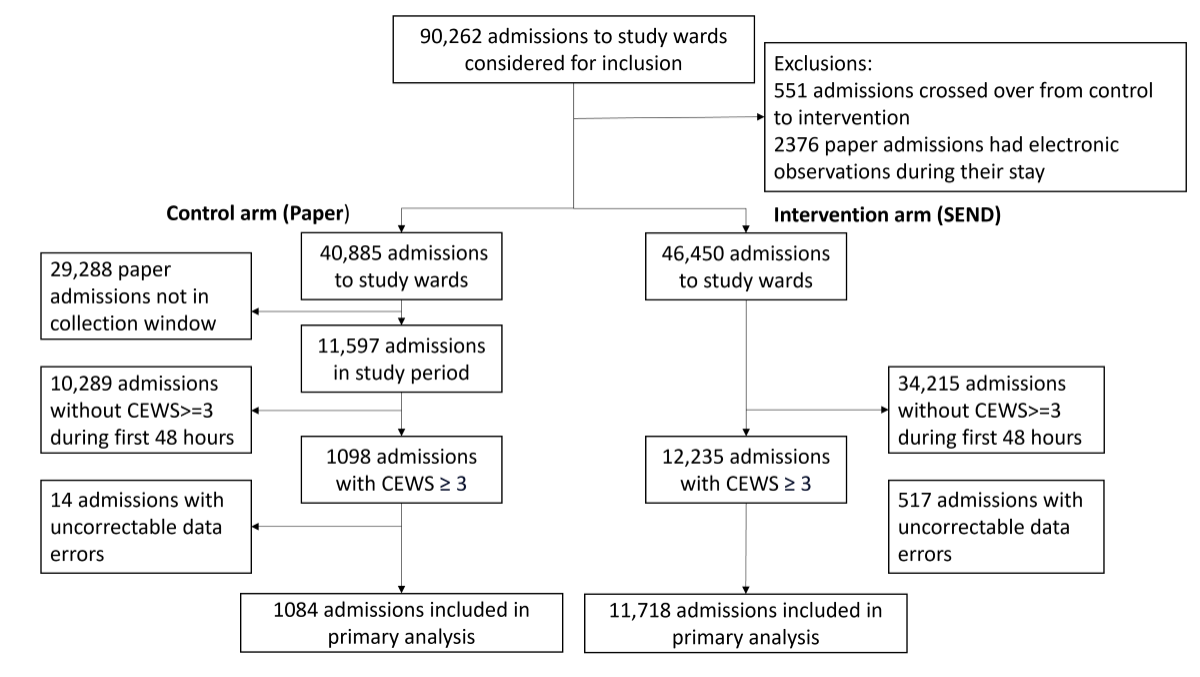
Recruitment flow diagram showing how patient admissions were recruited to this stepped-wedge study. Assignment to the Control or Intervention arm was determined by whether the admitting ward was using the SEND system, which followed the rollout schedule in Figure 2. The imbalance between arms is due to the limited data collection period (shown in Figure 2) prior to switching to SEND. CEWS: centile early warning score; SEND: system for electronic notification and documentation.

We conducted the study between January 2015 and September 2016, after the conclusion of the rollout of SEND. During this time, there were 90,262 admissions to the study wards. For 2927 (3%) of admissions, vital signs were recorded on both paper and SEND systems and thus excluded. Of the remaining 87,335 admissions, 40,885 (47%) had vital signs recorded exclusively on paper (control arm) and 46,450 (53%) admissions involved patients who had vital signs recorded exclusively using SEND (intervention arm). Of the admissions in the control arm, 11,597 occurred during the implementation period and were available for data capture. In total, 12,802 admissions were entered into the analysis, consisting of 1084 admissions in the control arm and 11,718 admissions in the intervention arm that had a triggering observation within 48 hours of arrival on their first study ward (Figure 3).

Admission characteristics for the control and intervention are presented in Table 2. Admissions in the intervention arm tended to be slightly older (median age 65 vs 70 years), more likely to be male (49.3% vs 45.6%), and have a higher number of comorbidities (median Charlson score 3 vs 4).

**Table 2:** Baseline characteristics for the study population, which included all hospital admissions in “study wards” to the Oxford University Hospitals Foundation Trust between January 2015 and September 2016. Study wards included all adult wards across the Trust, except for the obstetric wards, emergency departments, day units, high dependency units, intensive care units, and investigation suites.

Characteristics			Control (paper)		Intervention (SEND^a^)
Admissions			1084		11,718
Patients			1048		10,708
Age (years), median (IQR)			65 (49-79)		70 (54-81)
Sex (male), n (%)			494 (45.6)		5777 (49.3)
Charlson score, median (IQR)			3 (0-10)		4 (0-12)
**Admission type, n (%)**					
	Elective	392 (36.2)		4281 (36.5)	
	Emergency	692 (63.9)		7427 (63.4)	
	Other	0 (0)		10 (0.1)	
**Admitting specialty, n (%)**					
	Medical	430 (40)		5618 (47.9)	
	Surgical	645 (59.5)		5894 (50.3)	
	Other	9 (0.8)		206 (1.76)	

^a^SEND: system for electronic notification and documentation.

The proportion of male to female sex in both study arms was similar across all steps apart from cluster 1, in which there were a small number of admissions on paper (n=10). There were no males in cluster 20, a cluster that contained only obstetrics and gynecology wards. Proportions of elective and emergency admissions, and medical and surgical admissions, were similar for each study arm across all clusters.

### Primary Outcome

There was no significant difference in the TTNO between the 2 arms after adjustment for competing events (Table 3). The median TTNO in the control arm was 128 (IQR 73-218) minutes. The median TTNO in the observation arm was 131 (IQR 73-223) minutes. The hazard ratio of the TTNO using paper charting and the TTNO using SEND was 0.99 (95% CI 0.91-1.07, *P*=.73). All model variants in the sensitivity analysis gave results consistent with the Hussey and Hughes model primary analysis. The numbers of each type of competing events in each arm are shown in Table 4.

**Table 3:** Hazard ratio for time to next observation (TTNO) after an initial early warning score of 3 or greater. A hazard ratio <1 implies that the TTNO was shorter in the control (paper) arm and >1 implies that the TTNO was shorter in the intervention (system for electronic notification and documentation) arm. There was no significant difference in TTNO using the Hussey and Hughes model, or any other variants.

Model		Hazard ratio (95% CI)	*P* value
**Hussey and Hughes model**		0.99 (0.91-1.07)	.73
	Time by strata interaction (FE^a^)	Does not fit	—^b^
	Time by cluster interaction (RE^c^)	0.98 (0.91-1.07)	.72
	Treatment by strata interaction (FE)	0.96 (0.83-1.12)	.63
	Treatment by cluster interaction (RE)	0.99 (0.90-1.07)	.73
	Treatment by time interaction (FE)	Does not fit	—

^a^FE: Fixed Effects.

^b^Not available.

^c^RE: Random Effects.

**Table 4:** Number of competing events in the control and intervention arms. The hospital admissions in both study arms had similar percentages of deaths, intensive care unit admissions, theatre admissions, and arrest calls.

Competing events	Control (paper), n (%)	Intervention (SEND^a^), n (%)
Death	50 (5)	826 (7)
ICU^b^ admission	22 (2)	237 (2)
Theatre admission	181 (14)	1508 (12)
Arrest call	4 (<1%)	44 (<1%)

^a^SEND: system for electronic notification and documentation.

^b^ICU: intensive care unit.

**Figure 4:**
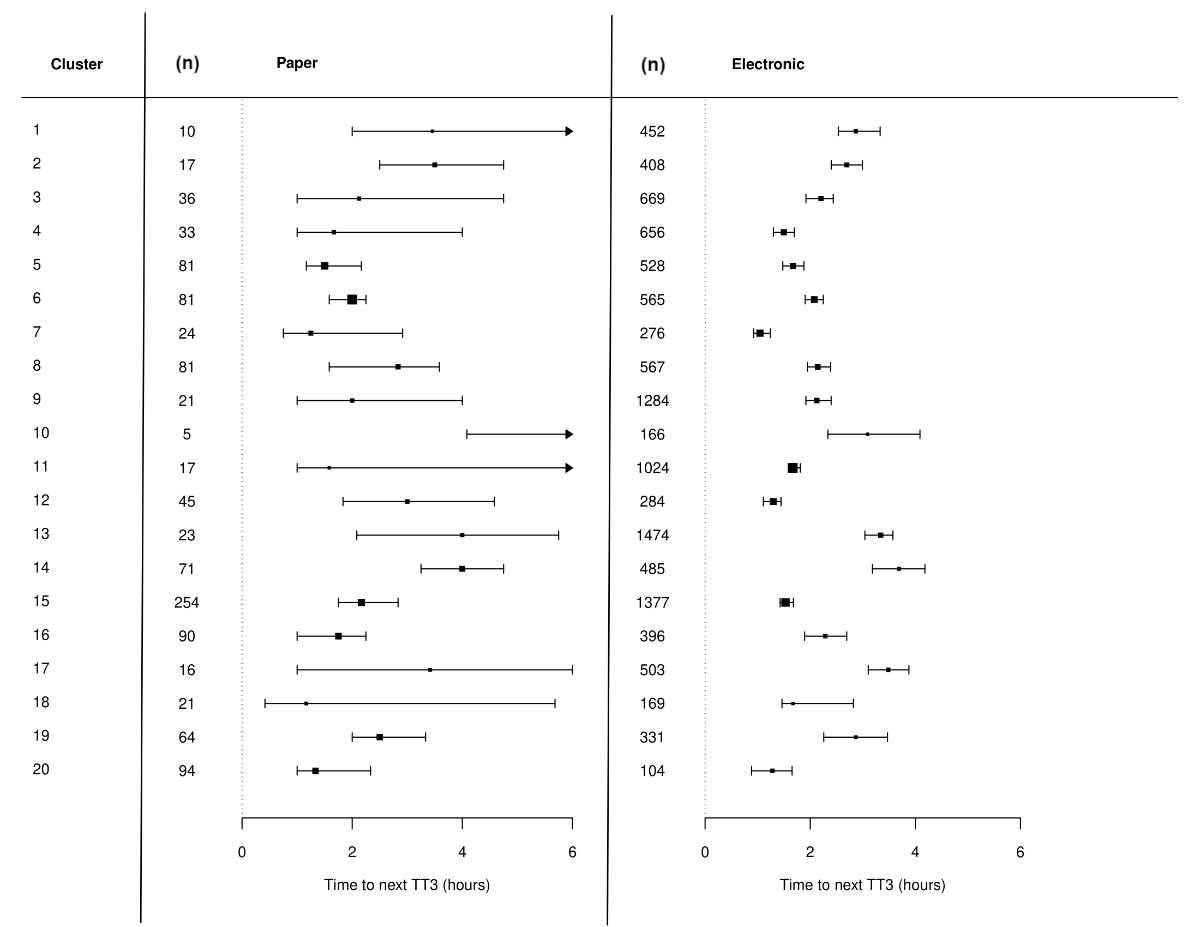
Time to next observations after an initial early warning score of 3 or greater by cluster for the control arm (left) and intervention arm (right). There was insufficient power to determine a statistically significant effect at the cluster level, as evidenced by overlapping confidence intervals between the left and right columns.

Figure 4 shows the TTNO for each step during the study. Confidence intervals for the electronic arm were much narrower than the electronic arm because there was more electronic data (collected after the initial intervention rollout period). There was a marked variation in the TTNO according to cluster (Figure 4); the introduction of the digital system did not reduce this variance. There was insufficient power to determine if the intervention had an impact at a cluster level. However, we note that there appeared to be a large reduction in TTNO for cluster 12, which were acute general medicine wards.

### Secondary Outcomes

The introduction of SEND had no significant effect on time to death in hospital, LOS, or time to unplanned ICU admission for the cohort included in the primary analysis (Table 5). There were only 48 cardiac arrest calls across the 2 arms of the study, therefore, there were insufficient events to model this outcome. The findings were consistent irrespective of modeling assumptions. Sensitivity analyses are reported in Multimedia Appendix 5.

**Table 5:** Hazard ratio for secondary outcomes. A hazard ratio of <1 implies that the outcome was shorter in the control (paper) arm and >1 implies that the outcome was shorter in the intervention (system for electronic notification and documentation) arm. None of the secondary outcomes were statistically significant at the *P*=.05 level.

Outcome	Hazard ratio (95% CI)	*P* value
Time to death in hospital	0.96 (0.68-1.36)	.84
Time to ICU^a^ admission	1.85 (0.98-3.49)	.06
Hospital length of stay	0.99 (0.65-1.51)	.97

^a^ICU: intensive care unit.

We also calculated the same secondary measures for the entire patient population (11,597 control and 46,450 intervention), including all those who did not score a CEWS ≥3 within the first 48 hours of admission (Multimedia Appendix 6). For this population, the start time was taken to be the time of admission to the study ward. In this group, there were no significant differences in time to death or LOS. However, there was a borderline reduction in time to ICU admission from the initial triggering set of observations in the intervention arm (hazard ratio 1.25, 95% CI 1.02-1.54).

### Usability

System usability scores were only available from Hospital A. The feedback questionnaire was sent to 1891 users, of which 208 (11%) responded. The system usability score was 77.6.

## Discussion

### Principal Findings

In this large, stepped wedge trial conducted across 4 hospital sites of the same National Health Service trust, the introduction of a digital charting system did not affect the frequency of vital signs recording, nor was it associated with changes in hospital mortality, cardiac arrest rates, or hospital LOS within the subgroup of patients who had a triggering EWS.

Our findings contrast with previous studies of digital vital signs charting. Jones et al [26] reported a reduction in the mean LOS from 9.7 to 6.9 days following the introduction of Patientrack (Alcidion Group Ltd). Schmidt et al [15] reported a reduction in hospital mortality following the introduction of VitalPAC (System C Healthcare Ltd).

The differences between our findings and those of previous researchers may be related to trial design and statistical analysis. A significant strength of our work is the use of a stepped-wedge trial design and a large data set, in line with international recommendations regarding digital health evaluation [27]. Furthermore, we did not institute any new clinical workflows when implementing SEND, which would have confounded the results.

Beyond issues related to design and analysis, 4 other hypotheses could explain our findings. First, it might be that the design or usability of SEND meant that nurses did not engage with the system. However, the system has previously been shown to be more efficient than the charting on paper and the score of 77.6 on the system usability scale is representative of good usability [28,29].

A second possibility is that, although the system was well-liked by staff, advice was not presented at the right time or in the right context and was therefore ineffective in reminding nurses to recheck vital signs [30]. Advice from the hospital protocol was presented at the time of observation recording but there was no mechanism for automatically notifying staff that the next set of observations was due and our implementation did not include the display of the time to the next observations on a dedicated screen at the nursing station. The understanding of how digital systems influence behavior is poorly understood.

A third possibility is that the system was effective in reminding nurses to recheck observations more frequently, but that the reminder alone was insufficient to trigger behavior change. Behavior change requires a combination of capability, opportunity, and motivation [31]. Even if a digital charting system positively alters motivation (through user prompts) and capability (through increased efficiency), these influences may be nullified by competing demands.

Finally, there is the possibility that, even with an effective reminder and supportive context, nurses were exercising clinical judgment and deliberately choosing to deviate from the hospital protocol. The gap between hospital protocols (“work as imagined”) and routine clinical practice (“work as done”) is well recognized and is often an essential adaptation to ensuring that hospitals continue to function [32]. While the hospital protocol recommended the same frequency of monitoring for all patients with an EWS greater than or equal to 3, our results showed that nurses increased the frequency of vital signs monitoring with the EWS score. It is possible that increasing the frequency of vital signs recording would not improve patient outcomes and rather than the nurses changing practice to match the hospital protocol, the protocol should be changed to match nursing practice more closely.

An unexpected finding was that when including all patients, irrespective of whether they had a triggering observation, the time to ICU admission in the intervention arm was less than in the control arm. Similar reductions in time to ICU transfer have recently been observed in a pre and postintervention study of a digital EWS system that used the electronic Cardiac Arrest Risk Triage EWS [33]. The difference was observed without any difference in the primary outcome measure, which might be explained in 2 ways. Either the result may not correspond to a true effect (which is consistent with the associated wide confidence intervals), or else SEND may be exerting effects via a mechanism other than increased frequency of patient observations.

### Limitations

The primary limitation of the study design was that clusters were not randomized but were instead determined by the predetermined phased rollout plan for SEND. Lack of randomization may be a problem since the estimate of the treatment may be unbiased if secular trends exist. To mitigate against this, we included a large number of clusters and explored a variety of analysis methods to examine the possibility of a secular trend. The stepped approach retains advantages over a simple before-after design. The presence of a control group available throughout the study period means that system-level changes may be detected.

A further limitation was the relatively small number of secondary end points. This led to instances in which some clusters had zero secondary end point events. Therefore, conclusions from the secondary outcome analysis ought to be interpreted with caution.

Caution is also required in interpreting the usability survey results. In our original study protocol, we had intended to obtain system usability score data from all new users of the system at the end of roll-out to each hospital site. However, flaws in our survey administration procedures inhibited us from identifying new users versus clinical users who worked in multiple hospitals. Therefore, we only surveyed users of the first site. It is possible that they were not representative of all users. Furthermore, there may be responder bias associated with the low response rate. However, the results obtained in this study are consistent with the findings of questionnaires from staff on pilot wards during the SEND development process [28].

Although data in this study were collected in 2016, we emphasize that the findings remain highly relevant to both the United Kingdom and international health care providers. In the United Kingdom, digital EWS systems are not yet ubiquitous and have been implemented at multiple hospital Trusts in the last year [34,35]. Internationally, the use of both EWS and an accompanying digital system is an emerging practice [36]. More pertinently, the effectiveness of EWS and the mechanism by which any potential benefits are obtained is still an open question. Indeed, a recent pre- and postevaluation of a digital sepsis score system highlighted the ongoing need for understanding how the use of alert systems evolves over time and impacts clinical workflow [37].

Finally, the findings presented here likely underestimate the true overall benefit of the system. We only examined the effects of SEND using a single measure of observation recording practice, the time between observations, is primarily a reflection of the impact of the system on nursing processes. We did not examine the impact of SEND on other clinical processes or the benefits of secondary use of the data for clinical governance and research.

### Conclusion

The introduction of a digital vital signs charting system had no effect on the frequency of vital signs observation or the time to ICU admission, hospital LOS, and hospital mortality in patients with a high EWS. Our findings stand in contrast to previous claims that the introduction of a digital vital signs charting system is associated with significant improvement in clinical outcomes. Future research should continue to investigate the mechanisms by which digital vital signs charting systems alter staff behaviors and improve patient outcomes.

## Appendix

Multimedia Appendix 1 EWS chart and escalation protocol.

Multimedia Appendix 2 Dates of steps.

Multimedia Appendix 3 System Usability Scale questionnaire adapted for SEND.

Multimedia Appendix 4 Power calculation.

Multimedia Appendix 5 Sensitivity analysis.

Multimedia Appendix 6 Secondary outcomes for the entire patient population (11,597 control and 46,450 intervention), including all those who did not score a CEWS≥3 within the first 48 h of admission.

## Abbreviations

CEWS: centile early warning score
EWS: early warning score
ICU: intensive care unit
LOS: length of stay
OUHFT: Oxford University Hospitals Foundation NHS Trust
SEND: system for electronic notification and documentation
TTNO: time to next observation

## Data Availability

The data sets generated and analyzed during this study are not publicly available as they are recorded at the patient level, such that it might be possible to reidentify individuals. They are available from the corresponding author on reasonable request.
