# Supplementary figures and images for: Effect of Digital Early Warning Scores on Hospital Vital Sign Observation Protocol Adherence: Stepped-Wedge Evaluation
Source: J Med Internet Res. 2024 Jun 20;26:e46691. doi: 10.2196/46691 (PMC11224703; doi:10.2196/46691)

## A – EWS chart and escalation protocol


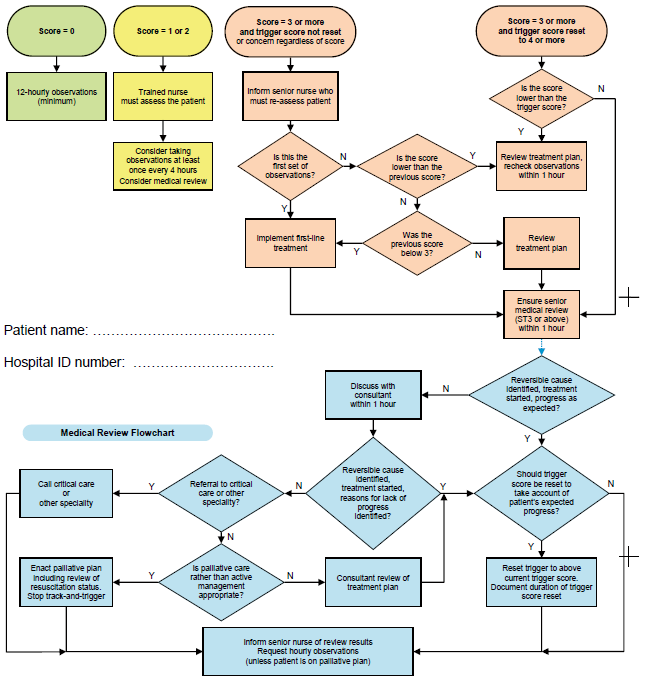

Supplement: Multimedia Appendix 1 [file jmir_v26i1e46691_app1.docx]
