# Supplementary material for: Effect of Digital Early Warning Scores on Hospital Vital Sign Observation Protocol Adherence: Stepped-Wedge Evaluation
Source: J Med Internet Res. 2024 Jun 20;26:e46691. doi: 10.2196/46691 (PMC11224703; doi:10.2196/46691)
Supplement: Multimedia Appendix 2 [file jmir_v26i1e46691_app2.docx]

## B – Dates of steps

| **Step** | **Date of rollout** |
| --- | --- |
| 1. Hospital A, Cluster 1 (1 Ward) | 09-Feb-2015 |
| 1. Hospital A, Cluster 2 (1 Ward) | 23-Feb-2015 |
| 1. Hospital A, Cluster 3 (1 Ward) | 09-Mar-2015 |
| 1. Hospital A, Cluster 4 (2 Wards) | 23-Mar-2015 |
| 1. Hospital A, Cluster 5 (1 Ward) | 06-Apr-2015 |
| 1. Hospital A, Cluster 6 (1 Ward) | 20-Apr-2015 |
| 1. Hospital A, Cluster 7 (1 Ward) | 04-May-2015 |
| 1. Hospital A, Cluster 8 (1 Ward) | 18-May-2015 |
| 1. Hospital B, Cluster 1 (4 Wards) | 22-Jun-2015 |
| 1. Hospital B, Cluster 2 (1 Ward) | 06-Jul-2015 |
| 1. Hospital C, Cluster 1 (3 Wards) | 12-Oct-2015 |
| 1. Hospital C, Cluster 2 (2 Wards) | 02-Nov-2015 |
| 1. Hospital D – Main Wing, Cluster 1 (4 Wards) | 07-Dec-2015 |
| 1. Hospital D – Main Wing, Cluster 2 (4 Wards) | 18-Jan-2016 |
| 1. Hospital D – Main Wing, Cluster 3 (5 Wards) | 08-Feb-2016 |
| 1. Hospital D – Main Wing, Cluster 4 (2 Wards) | 29-Feb-2016 |
| 1. Hospital D – 2nd Wing, Cluster 1 (2 Wards) | 06-Jun-2016 |
| 1. Hospital D – 2nd Wing, Cluster 2 (1 Ward) | 27-Jun-2016 |
| 1. Hospital D – 2nd Wing, Cluster 3 (1 Ward) | 18-Jul-2016 |
| 1. Hospital D – 2^nd^ Wing, Cluster 4 (1 Ward) | 30-Aug-2016 |
