# Supplementary material for: Effect of Digital Early Warning Scores on Hospital Vital Sign Observation Protocol Adherence: Stepped-Wedge Evaluation
Source: J Med Internet Res. 2024 Jun 20;26:e46691. doi: 10.2196/46691 (PMC11224703; doi:10.2196/46691)
Supplement: Multimedia Appendix 3 [file jmir_v26i1e46691_app3.docx]

## D – SUS questionnaire

1. Please choose the job title which best describes your role.
   {Nurse/HCA, Doctor, Allied Health Professional, Non-clinical}
2. Please select the ward where you predominantly use the SEND application. If you work on multiple wards equally please choose the ward on which you first started using SEND regularly.
   {List of wards}
3. How many years have you worked within the NHS or equivalent healthcare system?
4. How often do you use SEND to help deliver patient care?
   (Daily, At least once a week, At least once a month, Less than once a month, I don’t use SEND to deliver patient care}
5. Do you feel that you have received adequate training in how to use SEND?

   *SUS Questions*

- *Answered on a 5-point Likert scale ranging from Strongly Disagree to Strongly Agree*

1. I think that I would like to use SEND frequently.
2. I found SEND unnecessarily complex.
3. I thought SEND was easy to use.
4. I think that I would need assistance to be able to use SEND.
5. I found the various functions in SEND were well integrated.
6. I thought there was too much inconsistency in SEND.
7. I would imagine that most people would learn to use SEND very quickly.
8. I found SEND very cumbersome/awkward to use.
9. I felt very confident using SEND.
10. I needed to learn a lot of things before I could get going with SEND.

    *Additional questions*
11. Please describe how having SEND in your clinical area has affected the care you are able to deliver to patients.
12. Please describe any aspect of SEND training, installation or maintenance that has affected your ability to care for patients
13. Any other comments or suggestions?
14. If you would like us to respond to your comments directly please enter your email address. We will not use your email address for any other purpose.
