# Supplementary material for: Effect of Digital Early Warning Scores on Hospital Vital Sign Observation Protocol Adherence: Stepped-Wedge Evaluation
Source: J Med Internet Res. 2024 Jun 20;26:e46691. doi: 10.2196/46691 (PMC11224703; doi:10.2196/46691)
Supplement: Multimedia Appendix 4 [file jmir_v26i1e46691_app4.docx]

## C – Power Calculation

### Description of the Data Used for Analysis

Data from these analyses came from the CALMS-2 Dataset

16166 transcribed sets of observations with a time stamp

921 obs sets had a time stamp but did not have scores transcribed

207 individual patients (therefore 207 sets of obs where time to next obs could not be calculated)

### Data excluded from analysis

14610 pairs of obs sets used for analysis

359 obs were taken within 15 minutes after the previous ones. Excluded as these likely represent rechecks.

86 obs were taken after a gap of 12hrs or more. Excluded as these likely represent departure from the ward.

921 obs pairs where the first score wasn’t transcribed

### Current Nursing Practice

Trust protocol dictates that if the T&T score is ≥3 then the obs should be rechecked within 1 hour

160 patients (77%) had at least one T&T score ≥3

|  | Score <3 | Score≥3 | Total observations |
| --- | --- | --- | --- |
| Next Obs ≤1hr | 1564 (Over-achieving) | 587 | 2151 |
| Next Obs >1hr | 11606 | 853 (Failing) | 12459 |
| Total observations | 13170 | 1440 | 14610 |

The distribution of time differences for the Failing cohort is shown in Figure 1.

30% of the Failing cohort have their next observations taken within 2hrs. These might represent patients where the nurse recognised the importance of repeated observation but there were unavoidable delays. 5% had their next observation over 8hrs after the first triggering set. This are likely to represent patients who underwent a procedure or temporarily left the ward.


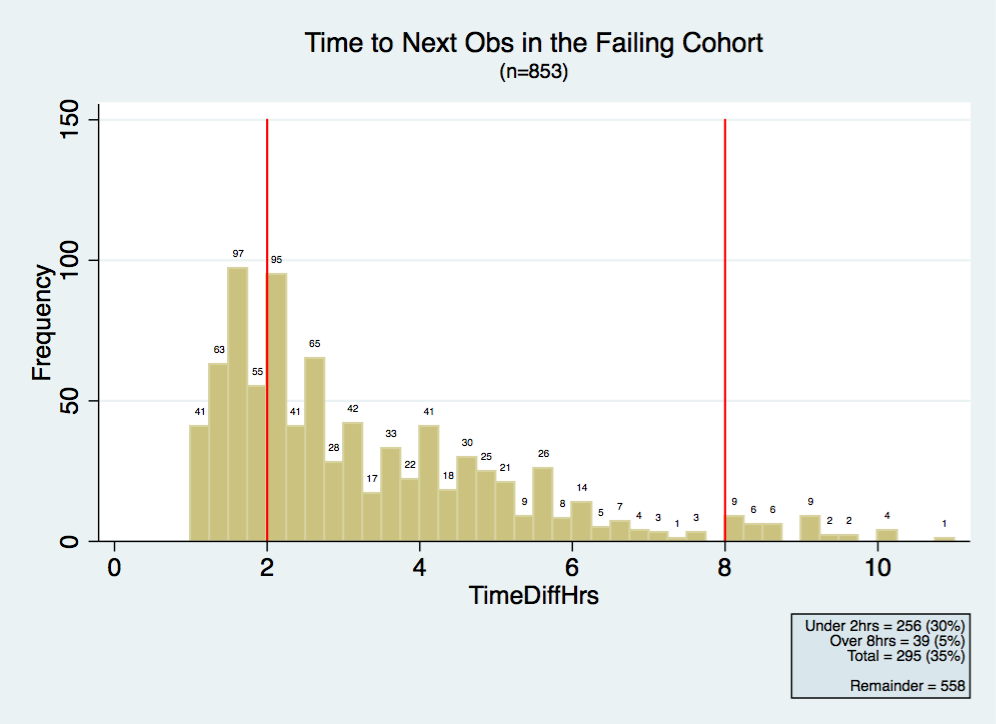


*Fig. 1*

### Is Nursing Behaviour Different for the First Observation Set that Triggers?


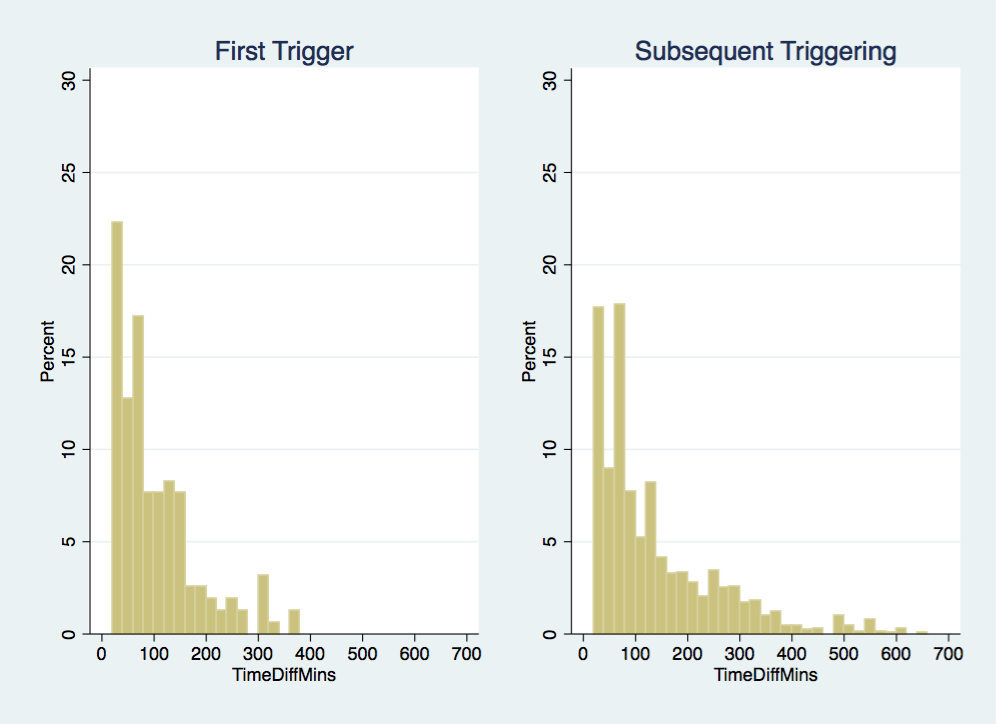


*Fig. 2*

Figure 2 shows the distributions of time to next obs. On the left is the time to next observations for the first observation set that scores ≥3 (n=157). On the right is the time to next observations for all other observation sets that scores ≥3 (n=1283).

## Sample Size Calculations

### Descriptors of Sample Where First Score ≥ 3

Variable | Obs Mean Std. Dev. Min Max

-------------+--------------------------------------------------------

timediffhrs | 1440 2.186146 1.915738 .3333333 10.83333

### Hypotheses about the effect of SEND

1. Time to take observations is determined by the following factors:
   1. Identification that patient has breached threshold and knowledge that next obs needed within 1 hour
   2. The number of competing tasks to be carried out
   3. Perceived importance of repeat observations, determined by
      1. Underlying attitudes
      2. End-of-the-bed appearance of the patient
2. 1.b and 1.c are probably the key determinants
3. SEND will affect 1.a and possibly, depending on the nature of the intervention, 1.c.i. As such the effect of SEND may either be modest across the board or restricted to a subset of the observations taken

| ***Evidence to support assertion 1.c.ii*** |
| --- |
| \|  \| Next Score <3 \| Next Score ≥3 \| Totals \| \| --- \| --- \| --- \| --- \| \| First Score <3 \| 12421 \| 749 (Worsening) \| 13170 \| \| First Score ≥3 \| 644 (Improving) \| 796 \| 1440 \| \| Totals \| 13065 \| 1545 \| 14610 \|   If your score is high then you have a 644/1440 (45%) chance of falling back under the threshold at next obs.  Of the 853 in the Failing cohort, 547 (64%) improved to under the threshold when their obs were rechecked  If your score is low then you have a 749/13170 (6%) chance of deteriorating  Of the 1564 in the Over-achieving cohort , 169(11%) subsequently deteriorated when obs were rechecked |

Based on the hypotheses above here are some possible scenarios.

For the 853 observations where the hospital protocol was not met

- If we reduced the time to take all obs by 30 mins then the mean would change by 42mins
- If we reduce the time to take all obs by 25% then the mean would change by 48 mins
- If we reduce the time to next obs by 1 hour for all obs where the delay is >2hrs but <8hrs then the mean would change by 36 mins
- If we reduce the time to next obs by 2 hours for all obs where the delay is >3hrs but <8hrs then the mean would change by 42 mins

Based on these a desirable difference in the means to detect would be between 30 and 60 mins. Using the graph in Figure 3

- To detect a difference of 30 mins in the mean time to take the next observation, following a T&T score of at least 3, would require a sample size of around 700 observation pairs (+15% as data are not normally distributed = ~800).
- To detect a difference of 45 mins in the mean time to take the next observation, following a T&T score of at least 3, would require a sample size of around 200 observation pairs (+15% as data are not normally distributed = ~230).
- To detect a difference of 1 hour in the mean time to take the next observation, following a T&T score of at least 3, would require a sample size of around 120 (+15% as data are not normally distributed = ~140).

The number of obs charts to view depends on whether each patient is allowed to contribute more than 1 instance of T&T score ≥3. In this dataset 77% of patients had at least 1 score of ≥3 and per patient the median number of observation sets scoring ≥3 was 6 (see Fig 4). These are probably higher than in the general wards.

In OTEST 50% of patients had at least 1 triggering score. Based on this if we assume that patients who trigger will have 4 observation sets which trigger then the number of charts to inspect becomes half the sample size (no charts = (sample size/4) /50%


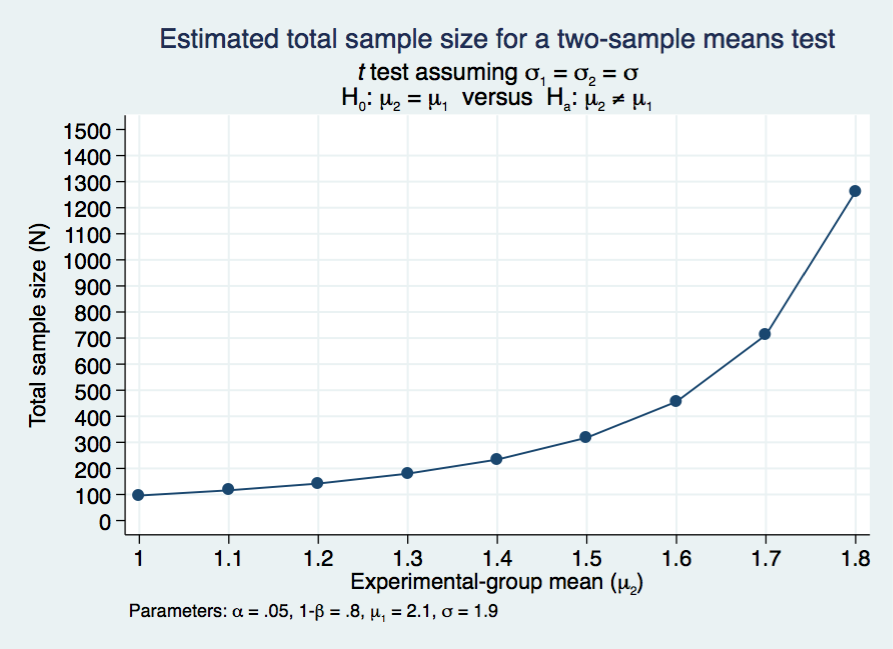


*Fig. 3*


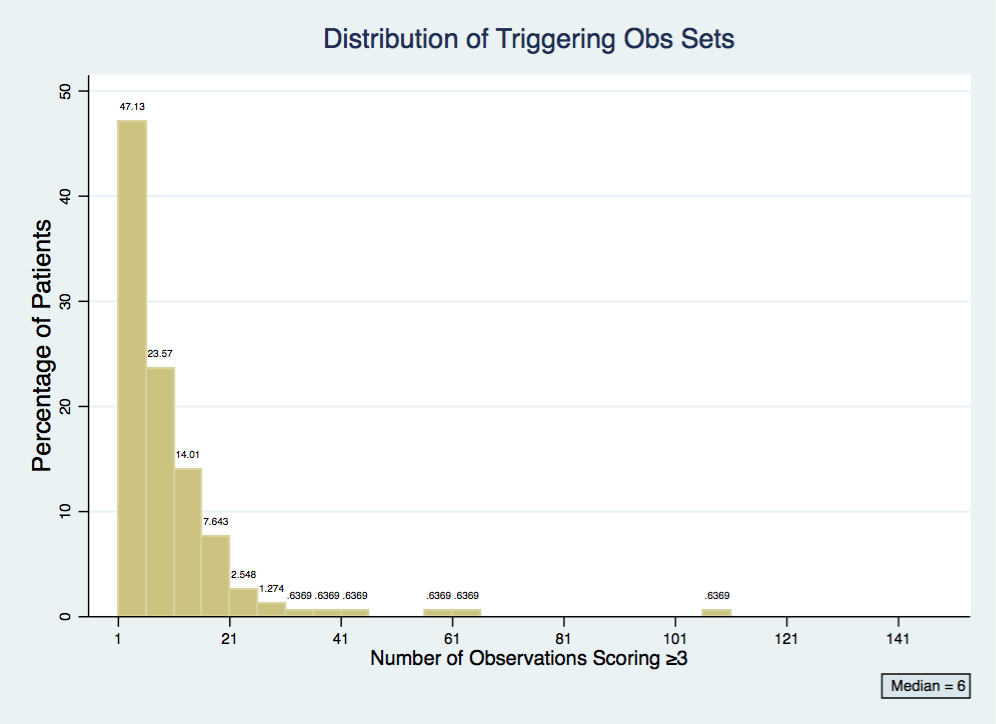


*Fig. 4*

### Run Length as an Endpoint

Under half the patients had at least one run

| **Number of Runs** | **Number of Patients** |
| --- | --- |
| 0 | 100 |
| 1 | 38 |
| 2 | 25 |
| 3 | 17 |
| 4 | 13 |
| 5 | 2 |
| 6 | 5 |
| 7 | 2 |
| 8 | 1 |
| 10 | 1 |
| 12 | 2 |
| 18 | 1 |

Only about 25% of patients had a run length that is long enough that we might reasonably make a difference.

| **Run Length** | **Number of Patients** |
| --- | --- |
| 0 | 100 |
| 2 | 35 |
| 3 | 19 |
| 4 | 19 |
| 5 | 7 |
| 6 | 7 |
| 7 | 5 |
| 8 | 4 |
| 9 | 2 |
| 11 | 5 |
| 12 | 1 |
| 13 | 2 |
| 33 | 1 |

Based on the plot in Figure 5, to find patients who have runs of a significant length we probably need people who have had at least 50 obs sets taken, which does not seem feasible.


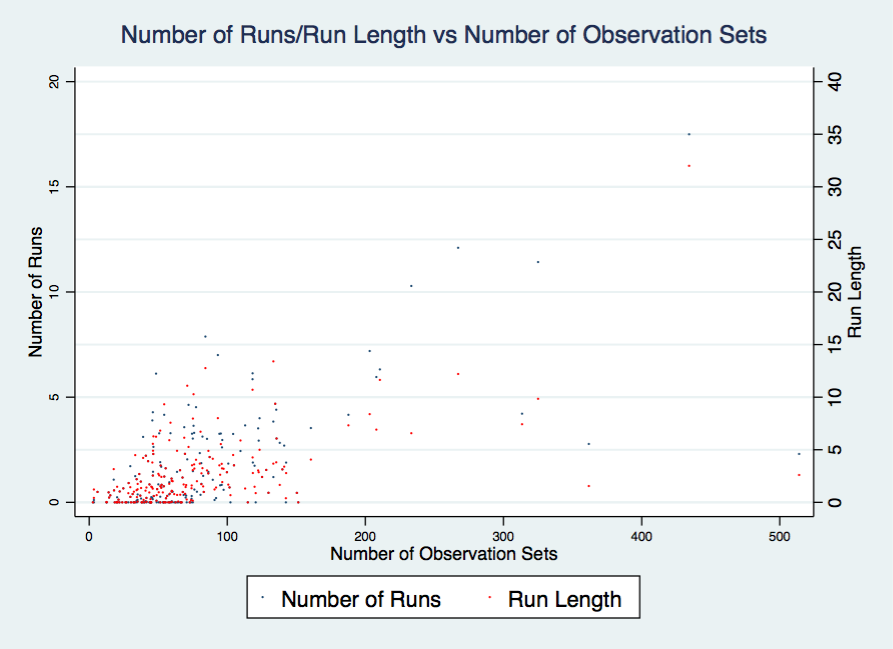


*Fig. 5*
