# Supplementary material for: Effect of Digital Early Warning Scores on Hospital Vital Sign Observation Protocol Adherence: Stepped-Wedge Evaluation
Source: J Med Internet Res. 2024 Jun 20;26:e46691. doi: 10.2196/46691 (PMC11224703; doi:10.2196/46691)
Supplement: Multimedia Appendix 5 [file jmir_v26i1e46691_app5.docx]

## E – Sensitivity Analysis

### Primary outcome results – time to next observation

| Model | Hazard Ratio (95% CI) | P-value |
| --- | --- | --- |
| Basic Hussey and Hughes model | 0.99 (0.91, 1.07) | 0.76 |
| A: Time by strata interaction (FE) | Does not fit | - |
| B: Time by cluster interaction (RE) | 0.99 (0.91, 1.07) | 0.75 |
| C: Treatment by strata interaction (FE) | 0.96 (0.83, 1.11) | 0.61 |
| D: Treatment by cluster interaction (RE) | 0.99 (0.91, 1.07) | 0.76 |
| E: Treatment by time interaction (FE) | Does not fit | - |

A hazard ratio < 1 implies paper better, and > 1 implies electronic better.

### Mortality

| Model | Hazard Ratio (95% CI) | P-value |
| --- | --- | --- |
| Basic Hussey and Hughes model | 0.94 (0.66, 1.33) | 0.72 |
| A: Time by strata interaction (FE) | Does not fit | - |
| B: Time by cluster interaction (RE) | 1.01 (0.71, 1.42) | 0.98 |
| C: Treatment by strata interaction (FE) | Does not fit |  |
| D: Treatment by cluster interaction (RE) | 0.94 (0.66, 1.33) | 0.72 |
| E: Treatment by time interaction (FE) | Does not fit | - |

A hazard ratio < 1 implies electronic better, and > 1 implies paper better.

### ICU Admission

| Model | Hazard Ratio (95% CI) | P-value |
| --- | --- | --- |
| Basic Hussey and Hughes model | 2.07 (0.98, 4.38) | 0.06 |
| A: Time by strata interaction (FE) | Does not fit | - |
| B: Time by cluster interaction (RE) | 2.08 (0.98, 4.40) | 0.06 |
| C: Treatment by strata interaction (FE) | Does not fit |  |
| D: Treatment by cluster interaction (RE) | 2.07 (0.98, 4.39) | 0.06 |
| E: Treatment by time interaction (FE) | Does not fit | - |

A hazard ratio < 1 implies paper has shorter time to ICU admission, and > 1 implies electronic has shorter time to ICU admission.

### Length of Stay

| Model | Hazard Ratio (95% CI) | P-value |
| --- | --- | --- |
| Basic Hussey and Hughes model | 0.98 (0.64, 1.49) | 0.92 |
| A: Time by strata interaction (FE) | Does not fit | - |
| B: Time by cluster interaction (RE) | 1.07 (0.71, 1.62) | 0.74 |
| C: Treatment by strata interaction (FE) | Does not fit |  |
| D: Treatment by cluster interaction (RE) | 0.98 (0.64, 1.49) | 0.92 |
| E: Treatment by time interaction (FE) | Does not fit | - |

A hazard ratio < 1 implies paper has shorter stay, and > 1 implies electronic has shorter stay.
