# Supplementary material for: Effect of Digital Early Warning Scores on Hospital Vital Sign Observation Protocol Adherence: Stepped-Wedge Evaluation
Source: J Med Internet Res. 2024 Jun 20;26:e46691. doi: 10.2196/46691 (PMC11224703; doi:10.2196/46691)
Supplement: Multimedia Appendix 6 [file jmir_v26i1e46691_app6.docx]

## F – Secondary Outcomes for entire patient population (11597 control, 46450 intervention), including all those who did not score a CEWS≥3 within the first 48 hrs of admission

| **Outcome** | **Hazard Ratio (95% CI)** | **P-value** |
| --- | --- | --- |
| **Time to death in hospital** | 1.06 (0.89, 1.26) | 0.53 |
| **Time to ICU Admission** | 1.25 (1.02, 1.54) | 0.03 |
| **Hospital length of stay** | 1.06 (0.89, 1.26) | 0.53 |

Table 6: Hazard ratio for secondary outcomes using Hussey and Hughes model. A hazard ratio < 1 implies that the outcome was shorter in the control (paper) arm and >1 implies that the outcome was shorter in the intervention (SEND) arm.
